# Hysterectomy by vaginal-assisted natural orifice transluminal endoscopic surgery: Initial experience with twelve cases

**DOI:** 10.4274/jtgga.2017.0075

**Published:** 2018-03-01

**Authors:** Cihan Kaya, İsmail Alay, Murat Ekin, Levent Yaşar

**Affiliations:** 1 Clinic of Obstetrics and Gynecology, University of Health Sciences, Bakırköy Dr. Sadi Konuk Training and Research Hospital, İstanbul, Turkey

**Keywords:** Hysterectomy, laparoscopic surgery, natural orifice transluminal endoscopic surgery

## Abstract

**Objective:**

To declare our initial experience on hysterectomy cases performed using vaginally-assisted natural orifice transluminal endoscopic surgery.

**Material and Methods:**

The study was conducted with data from 12 patients in our department who were recommended for hysterectomies for various indications between January 2017 and May 2017. The following data were collected retrospectively: age, body mass index (BMI), parity, previous abdominal or pelvic surgery, total operating time, preoperative hemoglobin (Hb), postoperative Hb, peri-operative complications and Visual Analogue Scale scores for evaluating postoperative pain. All patients were laid in the dorsal lithotomy position under general anesthesia. A cervical circumcision, as well as anterior and posterior colpotomy were performed. A self-constructed glove port was then inserted through the anterior and posterior colpotomy openings into the abdominal cavity. After pneumoperitoneum was achieved, a 10-mm rigid zero-degree telescope, disposable conventional laparoscopic grasping forceps, and a tissue sealer were used as standard equipment. After exploration of the abdominal cavity, all uterine vessels and ligaments were sealed and transected using the tissue sealer. After removing the uterus, the vaginal opening was closed using a Vicryl 1-0 suture.

**Results:**

The following are the mean values for each variable: patients’ age: 55.75±9.8 years (range, 43-72 years), BMI: 29.4±5.4 kg/m^2^ (range, 21-42 kg/m^2^), operation duration: 66.8±25.3 min (range, 42-120 min), decrease in Hb: 1.5±1 (0-4) gr/dL, and hospital stay: 2.1±0.3 (2-3) days. There were no vaginal wound infections or dehiscence, and no patients reported pain during postoperative pelvic examinations.

**Conclusion:**

Although these findings are from our initial experience, we can affirm the feasibility of this technique.

## Introduction

Minimally invasive laparoscopic techniques have been widely used for gynecologic diseases. Advances in technological equipment such as flexible optics, reticulated graspers, and sealers have allowed umbilical single-port surgeries to become more common in daily practice. Single-site surgeries have benefits over conventional multiple-port laparoscopic surgeries, which include decreased anxiety from unintended surgical skin incisions and port site pain (1). In recent years, natural orifice transluminal endoscopic surgery (NOTES) has been used by general surgeons, urologists, and gastroenterologists for performing cholecystectomy, appendectomy or nephrectomy through natural body orifices such as the stomach, esophagus, bladder, rectum and vagina. It has also been reported that oophorectomies and even hysterectomies can be performed using NOTES by gynecologic surgeons (2). These procedures have the advantage of magnifying pelvic structures with optical systems, and thus increase the comfort of surgery through better visualization than open abdominal or vaginal surgeries. NOTES procedures also have the potential benefit of reduced umbilical or port site hernia rates and shorter recovery periods than conventional laparoscopic procedures (3).

Historically, the transvaginal route has been routinely used for diagnostic evaluation of infertility (4). Recently, a randomized controlled study was published by Baekelandt et al. (1) that compared transabdominal laparoscopy and NOTES. However, there is still a lack of knowledge on NOTES procedures in gynecologic practice. In this report, we aimed to declare our initial experience on hysterectomy cases that were performed with vaginal-assisted (Va) NOTES.

## Material and Methods

The study was conducted with data from 12 patients in our department who were recommended for hysterectomies for various indications between January 2017 and May 2017. All procedures were performed by the same experienced surgeon in endoscopy (C.K.); ethical approval was obtained from our institutional ethics committee (Approval number: 2017/04/24). Data analyses of all patients who gave informed consent were included. 

The inclusion criteria for patients undergoing VaNOTES hysterectomy were as follows: no contraindication for pneumoperitoneum or the Trendelenburg position, no fixed uterus or nodularity in the pouch of Douglas on bimanual pelvic examination, and no history of pelvic inflammatory disease, pelvic abscess or endometriosis. The following data were collected retrospectively: age, body mass index (BMI), parity, type of delivery, previous abdominal or pelvic surgeries, duration of anterior and posterior colpotomy, total operating time, preoperative hemoglobin (Hb), postoperative Hb, peri-operative complications, and decrease in Hb levels. 

The duration of anterior and posterior colpotomy was defined as from the beginning of cervical circumcision to the point of accessing the abdominal cavity via both anterior and posterior openings. The total duration of surgery was defined as the time from the placement of the Foley catheter to the end of vaginal closure. Peri-operative complications included bowel, bladder, ureteral or vascular injuries and blood loss >300 mL. Postoperative complications included lower urinary tract infections, ileus, bleeding from the vaginal cuff or infections, and pelvic hematoma. Postoperative pain scores were assessed using a Visual Analogue Scale (VAS) (scoring from 0=no pain to 10=worst pain ever) after 6 and 24 hours postoperatively. Prophylactic antibiotic therapy was induced with 2 g of cefazolin during surgery. Each patient was called for postoperative follow-up after one and four weeks after surgery. 

### Anterior and posterior colpotomy

The patients were laid in the dorsal lithotomy position under general anesthesia. After appropriate sterilization and draping of the surgical field, a Foley catheter was inserted into the urethra. The cervix was identified using vaginal retractors and grasped at the upper and lower lips with two tenaculum forceps. A cervical hydrodissection was performed with a 20 cc saline injection, after which cervical circumcision was performed with a 20-mm scalpel. For the anterior colpotomy, the vaginal mucosa and bladder were pushed up along the uterine cervical fascia using a small surgical gauze. Once the anterior peritoneum was identified, it was opened with scissors, and the same dissection procedure was performed to access the posterior peritoneum until the pouch of Douglas was reached.

### Insertion of self-constructed glove port

A size 8 surgical glove without powder was used. The middle finger of the surgical glove was incised with a 1-cm opening, through which a 10-mm disposable trocar was inserted for CO_2_ insufflation and an endoscopic camera. The index and the 4^th^ finger of the glove were incised with a 0.5-cm opening, through which two 5-mm disposable trocars were inserted for the laparoscopic instruments. All trocars were fixed to the glove using a number 0 silk suture. A small-sized Alexis Wound Protector/Retractor (Applied Medical, Rancho Santa Margarita, CA, USA), which was attached to the glove, as well as a self-constructed port, were inserted through the anterior and posterior colpotomy openings into the abdominal cavity.

### Laparoscopic procedure

**Figure 1:**
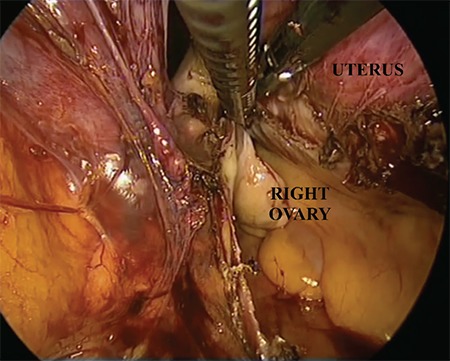
Endoscopic vaginal view of uterine cervix and corpus

**Figure 2:**
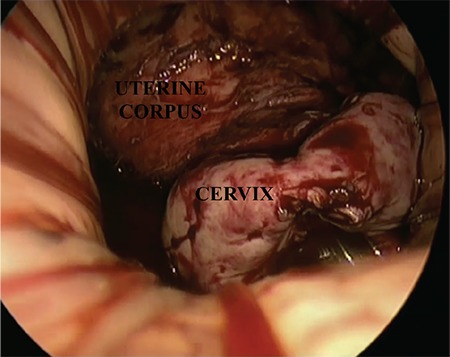
Endoscopic vaginal view of right salpingectomy procedure by using tissue sealer

After achieving pneumoperitoneum with 10 mm Hg CO_2_ insufflation, a 10-mm rigid zero-degree telescope was then inserted for optical imaging (Karl Storz visualization system; Karl Storz Tuttlingen, Germany). Disposable conventional laparoscopic grasping forceps and a tissue sealer (Enseal G2 articulating tissue sealer, Ethicon Endo Surgery, Cincinnati, OH, USA) were used as standard equipment. A suction-irrigation cannula was used when and where needed. After exploration of the abdominal cavity and pelvic organs, both sacro-uterine ligaments were sealed and transected using a tissue sealer. This procedure was repeated for the parametrium, both uterine arteries, ovarian ligaments and the Fallopian tube (Figure 1, 2). Both infundibulopelvic ligaments were sealed and transected if a bilateral salpingo-oopherectomy was planned. After complete resection of the uterus from its ligaments and vessels, it was placed into the glove (size permitting) or pulled into the abdominal cavity to obtain better visualization for hemostasis and rinsing of the peritoneal cavity. Afterwards, the pneumoperitoneum was deflated and the glove port was removed. The uterus and/or adnexa was extracted using tenaculum forceps. The vaginal opening was closed with a Vicryl 1-0 suture (Ethicon, Piscataway, NJ, USA).

## Results

Twelve VaNOTES hysterectomy procedures were performed and no conversion to standard multi-incision laparoscopy was needed. The following are the mean values for each variable: patients’ age: 55.75±9.8 years (range, 43-72 years), gravidity: 2.5±05 (2-3), parity: 2.5±05 (2-3), BMI: 29.4±5.4 kg/m^2^ (range, 21-42 kg/m^2^), uterus weight: 188.5±76.6 gr (100-300), size of uterus: 7.83±1.58 weeks (range, gestational week), operation duration: 66.8±25.3 (42-120) minutes, preoperative Hb: 12.1±1.3 (10-14) gr/dL, postoperative Hb: ±10.5 (9-13) gr/dL, decrease in Hb: 1.5±1 (0-4) gr/dL, blood loss: 170.83±68.95 cc (range, 100-300 cc), hospital stay: 2.1±0.3 (2-3) days, postoperative 6^th^ hour VAS score: 6.2±0.5 (5-7), postoperative 24^th^ hour VAS score: 2.7±0.6 (2-4). Five patients had bilateral salpingo-oophorectomy in addition to hysterectomy, and the remaining 7 patients had only bilateral salpingectomy in addition to hysterectomy. Table 1 presents an overview of the patient and perioperative data. Each patient was examined during the first and fourth weeks after surgery. There were no vaginal wound infections or dehiscence, and no patients reported pain during the postoperative pelvic examination.

## Discussion

Anatomically, the vaginal route is a well-known approach for surgical procedures by gynecologists. However, only a handful of institutions have started performing a transvaginal NOTES for benign gynecologic conditions (3,5,6). Current evidence is based on case series reported from these centers.

Baekelandt recently reported results of ten hysterectomies performed using transvaginal NOTES. In his study, there was no conversion to standard laparoscopy or laparotomy in any of the ten patients, with a mean operation time of 97 minutes (range, 60-120 min). He concluded that the NOTES approach may be useful for avoiding abdominal wall wounds and trocar-related complications (3). In another study by Lee et al. (5), the largest hysterectomy series yet with 137 patients undergoing the transvaginal NOTES technique, 94.9% (n=130) of patients were successfully treated, with the mean operative time and hospital stay being 88.2±4.1 minutes and 2.8 days, respectively. The mean uterine weight was 450.0±24.1 grams. Two patients had complications including intraoperative hemorrhage and cystotomy, and five had transvaginal colpotomy failure, cul-de-sac obliteration by bowel adhesions or mass obstruction. Another five patients had postoperative urinary retention or febrile morbidity (5). Yang et al. (6) reported 16 patients who underwent VaNOTES hysterectomies in comparison with 32 who underwent paired- and single-port laparoscopy-assisted vaginal hysterectomies (SP-LAVH). The authors reported no difference in intraoperative and postoperative outcomes such as estimated blood loss decrease in Hb on postoperative day 1, VAS scores, and febrile complications. However, the mean operative time was 70.6 minutes for VaNOTES hysterectomies and 93.2 minutes for SP-LAVH. The median postoperative hospital stay was 3.5 days for VaNOTES hysterectomies and 4 days for SP-LAVH (6). Jallad et al. (7) also recently reported eight salpingo-oophorectomy cases (six of them were unembalmed cadavers and two were live patients) in which the transvaginal NOTES technique was performed in the patients. First, they performed vaginal hysterectomy with a conventional technique and subsequently performed a salpingo-oophorectomy through a transvaginal NOTES technique. In this study, the ovaries appeared normal, and the main objective was to prove that transvaginal NOTES may facilitate salpingo-oophorectomy procedures after conventional vaginal hysterectomies.

In our study, we succeeded in finalizing the operations with a planned route using VaNOTES, with a mean operation duration that was lower than previously reported (3,5). This result may depend on the sealing device that we used for transection of the uterine attachments. In the studies mentioned above, the mean hospital stay varied between 2.8 to 3.5 days (3,5,6). On the other hand, the mean postoperative hospital in our study was merely 2.1 days, even after keeping patients as inpatients to observe any postoperative early complications. The mean Hb decrease and VAS scores in our study was similar to previous investigations.

Although these findings are from our initial experience, we firmly believe in the feasibility of this technique. By advancing the clinical experience of these cases, we may postulate that NOTES hysterectomies may take the place of total laparoscopic hysterectomies or single-port laparoscopic hysterectomies in the near future.

## Figures and Tables

**Table 1:**
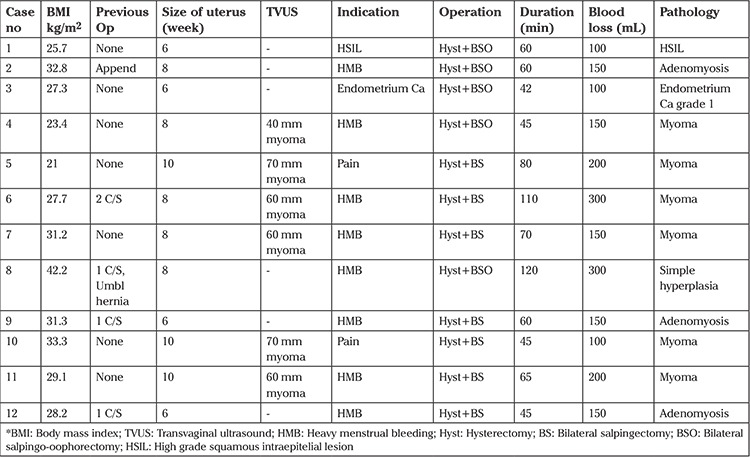
Overview of VaNOTES hysterectomy results and perioperative data
